# Identificación antropométrica del exceso de adiposidad en adultos argentinos según los nuevos conceptos de obesidad

**DOI:** 10.15446/rsap.V27n3.119461

**Published:** 2025-05-01

**Authors:** Martín Gustavo Farinola

**Affiliations:** 1 MF: Lic. Actividad Física y Deporte. M. Sc. Metodología de la Investigación Científica. Ph. D. Epistemología e Historia de la Ciencia. Departamento de Humanidades y Ciencias Sociales, Universidad Nacional de La Matanza. San Justo, Buenos Aires, Argentina. mfarinola@unlam.edu.ar Universidad Nacional de La Matanza Departamento de Humanidades y Ciencias Sociales Universidad Nacional de La Matanza Buenos Aires Argentina mfarinola@unlam.edu.ar

**Keywords:** Obesidad, antropometría, Argentina *(fuente: DeCS, BIREME)*, Obesity, anthropometry, Argentina *(source: MeSH, NLM)*

## Abstract

**Objetivo:**

Calcular la prevalencia del componente antropométrico elevado, según los nuevos conceptos de obesidad, en una muestra probabilística de la población adulta argentina.

**Métodos:**

Se utilizaron los resultados de la 4.a Encuesta Nacional de Factores de Riesgo Argentina. Se incluyeron sujetos de 18 a 65 años a quienes se les haya medido directamente peso, estatura y circunferencia de cintura (n=12 871 participantes de ambos sexos). Se calculó la prevalencia del componente antropométrico elevado para el diagnóstico de obesidad según los criterios tradicional (IMC >30), de la Asociación Europea para el Estudio de la Obesidad (EASO) y de un Comité Internacional de Expertos (CIE).

**Resultados:**

Las prevalencias obtenidas en ambos sexos estuvieron en el 31,8% con el criterio tradicional, entre 48,7% y 59,3% según criterio EASO y entre 32,7% y 39,0% según CIE. Las diferencias entre varones y mujeres fueron significativas.

**Conclusiones:**

Dada la diferencia de resultados entre los nuevos criterios antropométricos propuestos para el diagnóstico de obesidad, será necesario trabajar sobre la identificación de cuál de ellos representa mejor el exceso de adiposidad en adultos argentinos.

## Abstract (en)

**Objective:**

To calculate the prevalence of elevated anthropometric component according to new concepts of obesity in a probabilistic sample of Argentine adult population.

**Method:**

Results of the Argentine 4th Encuesta Nacional de Factores de Riesgo were used. Subjects aged 18 to 65 years who had their weight, height and waist circumference directly measured were included (n=12,871 participants of both sexes). Prevalence of elevated anthropometric component was calculated according to traditional criteria (BMI>30), European Association for the Study of Obesity (EASO) criteria and international committee of experts (ICE) criteria for the diagnosis of obesity.

**Results:**

Prevalences obtained in both sexes were 31.8% with the traditional criterion, between 48.7 and 59.3% according to EASO criterion, and between 32.7 and 33.7% according to the ICE. Differences between men and women were significant.

**Conclusions:**

Given the difference in results between the new anthropometric criteria proposed for the diagnosis of obesity, it will be necessary to work on identifying which of them best represents excess adiposity in Argentine adults.

Tradicionalmente, el índice de masa corporal (IMC) (peso/estatura^2^) ha estado omnipresente en los sistemas de diagnóstico de obesidad, siendo el valor de corte sugerido 30 kg/m^2^ o mayor 1-3. En Argentina, según este criterio, la prevalencia de obesidad en población mayor de 18 años se calculó en 32,4% 3.

Si bien el IMC ha correlacionado relativamente bien con la morbilidad y la mortalidad en estudios poblacionales 4-7, cuenta con carencias a la hora de evaluaciones de salud individuales 8,9. Por ejemplo, puede arrojar falsos positivos en el caso de personas con elevado desarrollo muscular 10,11 y falsos negativos en el caso de personas sin exceso de peso pero con elevada adiposidad 12.

Recientemente se han realizado dos propuestas de reforma de técnicas y criterios para identificar a una persona con obesidad. Una de ellas representa la visión de la Asociación Europea para el Estudio de la Obesidad (EASO, por sus siglas en inglés) 8 y la otra fue realizada por un comité internacional de expertos (CIE) cuya conformación buscó una representación equilibrada de las disciplinas médicas pertinentes y de las diferentes regiones del mundo 9. Ambas propuestas presentan aspectos en común y aspectos en los que difieren. Entre los primeros se encuentra que ambas añaden un criterio clínico, que se adiciona al antropométrico, para asumir que una persona presenta obesidad; por otro lado, ambas proponen agregar un segundo indicador de adiposidad corporal complementario al IMC y ambas sostienen que los valores de corte antropométricos deben asignarse al menos según el origen étnico de la persona evaluada. Estas modificaciones presentes en ambas propuestas buscan disminuir la sobre y subestimación de los casos de obesidad al mejorar la identificación tanto del exceso de adiposidad (no de peso corporal) como del compromiso de la salud física o mental y/o de la capacidad de realizar las actividades diarias debido a la obesidad.

En cuanto a las diferencias puede notarse que son diversas. En primer lugar, cuando los dos componentes, el antropométrico y el clínico, lo señalan, entonces se diagnostica obesidad en el caso de EASO y obesidad clínica en el caso del CIE. En este sentido, el CIE adiciona una categoría llamada obesidad preclínica que, resumidamente, se presenta cuando hay exceso de adiposidad (componente antropométrico elevado) pero sin compromiso de tejidos y órganos ni de la capacidad de realizar las actividades diarias (componente clínico) 9. Otra diferencia es que EASO propone dos criterios antropométricos mientras que CIE presenta uno solo (Cuadro 1). Por otro lado, EASO utiliza el índice cintura/talla (ICT) como único indicador antropométrico complementario al IMC, mientras que CIE menciona una serie de indicadores posibles que van desde medidas de superficie relacionadas con la obesidad abdominal (circunferencia de cintura, índice cintura/cadera e ICT, entre otros) hasta mediciones de grasa corporal total (impedancia bioeléctrica o absorciometría dual de rayos X) (Cuadro 1). Además, EASO presenta valores de corte puntuales tanto para IMC (los tradicionales 25 y 30) como para ICT, pero aclara que estos valores son apropiados para personas con ascendencia europea 6.

**Cuadro 1:** Nuevos componentes antropométricos para el diagnóstico de obesidad

EASO (ref. 8)	CIE (ref. 9)
IMC ≥ 30 kg/m² o IMC ≥ 25 kg/m² más ICT > 0,5 (valores de corte sugeridos para sujetos con ascendencia europea)	IMC igual o superior a los umbrales de obesidad establecidos según edad, género y etnia más otra medida antropométrica (CC, ICC, ICT, etc.) o medición de la grasa corporal con DEXA o BIA

EASO: Asociación Europea para el Estudio de la Obesidad; CIE: comité internacional de expertos; IMC: índice de masa corporal; ICT: índice cintura/talla; CC: circunferencia de cintura; ICC: índice cintura/cadera; DEXA: absorciometría dual de rayos X; BIA: impedancia bioeléctrica.

En cambio, el CIE no presenta ningún valor de corte en particular y recomienda que estos se construyan según género, edad y ascendencia étnica o país 9.

Más allá de las diferencias entre las propuestas de EASO y CIE, la distancia entre estas nuevas propuestas y lo que se solía utilizar hasta el momento para identificar la presencia de obesidad puede provocar que con estos nuevos criterios también cambien las prevalencias de obesidad en los países. Esta cuestión no ha sido estudiada hasta el momento de la escritura de este artículo y requiere de nuevas estadísticas o investigaciones 9.

El objetivo de este trabajo es utilizar los resultados de la 4.a Encuesta Nacional de Factores de Riesgo argentina (ENFR) para comenzar a explorar la presencia de obesidad en la población adulta argentina según los nuevos criterios de diagnóstico. En concreto, se buscará calcular el porcentaje de la población argentina adulta de 18 a 65 años de edad que cumpla con el componente antropométrico elevado según las nuevas propuestas de diagnóstico de obesidad. La decisión de dedicar el trabajo solo al componente antropométrico radica en que los datos de la ENFR los creemos insuficientes como para calcular también el componente clínico, para lo cual serán necesarios nuevos trabajos de campo.

## MÉTODOS

### Diseño

Se utilizó un diseño transversal y se trabajó con una fuente de datos secundaria. Los datos se tomaron de la 4.^a^ ENFR llevada a cabo por el Ministerio de Salud de la República Argentina (MSRA) entre los meses de septiembre y diciembre de 2018 13.

### Muestra

La 4.^a^ ENFR utilizó un diseño muestral probabilístico, multietápico y representativo de alcance nacional y urbano en la República Argentina. Se obtuvo información de personas mayores de 18 años de edad residentes en viviendas particulares de localidades urbanas de 5000 y más habitantes para el cuestionario y las mediciones físicas 3.

El tamaño de la muestra alcanzó a 49 170 viviendas para la aplicación del cuestionario o Paso 1 de la encuesta (31 426 hogares y 29 224 personas con respuestas), abarcando a todas las jurisdicciones del país. Para las mediciones físicas o Paso 2, se realizó una submuestra con el 75% de dichas viviendas (23 556 hogares y 16 577 personas con respuesta) 3.

Para el presente análisis se incluyeron a aquellos sujetos de 18 a 65 años que cuenten con resultados de mediciones antropométricas directas (no autorreportadas).

### Mediciones

Las mediciones físicas, Paso 2, incluyeron tensión arterial, peso, talla y circunferencia de cintura (cc). La talla de pie se midió con un tallímetro y el peso corporal con una balanza digital. La circunferencia de cintura se midió con una cinta antropométrica en el punto medio entre la cresta ilíaca superior y la última costilla. En la 4.^a^ ENFR esta medición se tomó directamente sobre la piel siempre que se pudo, de lo contrario se tomó por encima de un tejido fino, pero no de ropa gruesa o voluminosa. Luego se calcularon ICT (ICT [] = cc [cm] / talla [cm]) e IMC (IMC [kg/m^2^] = peso [kg] / talla [m]^2^).

Las mediciones físicas fueron realizadas por personal del MSRA, que recibió capacitación específica previa al trabajo de campo. Las características del instrumental utilizado para realizar las mediciones fueron avaladas por el MSRA 3.

### Componente antropométrico elevado

El componente antropométrico en los nuevos criterios de diagnóstico de obesidad busca identificar a las personas con exceso de adiposidad, que no necesariamente son personas con obesidad (véase introducción). Por este motivo, no se calculará la presencia de obesidad sino la presencia de componente antropométrico elevado (CAE) según criterios EASO y CIE (Cuadro 1).

En el presente trabajo el criterio EASO tendrá dos posibilidades. En EASO1 se aplicarán los valores de corte propuestos para personas de ascendencia europea: IMC ≥ 30 kg/m2 o IMC ≥ 25 kg/m^2^ más ICT > 0,500 8. En EASO2 se aplicarán los valores de corte identificados en adultos argentinos: en varones IMC ≥ 28,6 kg/m^2^ o IMC ≥ 25 kg/tf más ICT ≥ 0,559; en mujeres IMC ≥ 27,8 kg/m^2^ o IMC ≥ 25 kg/m^2^ más ICT ≥ 0,573 14. En EASO2, el punto de corte "25" para IMC se ha tomado de Busetto 6, dado que en el estudio de adultos argentinos los dos valores de corte que se identificaron para IMC han sido muy cercanos 14.

Para el criterio CIE también se ensayarán dos posibilidades. En CIE1 los valores de corte utilizados surgen de una de las opciones utilizadas hasta el momento en Argentina, en las que se adjudica riesgo "alto" de salud: IMC ≥30 kg/m^2^ en ambos sexos más cc=94-102 cm en varones y 80-88 cm en mujeres o IMC=25-29,9 kg/m^2^ en ambos sexos más cc>102 cm en varones y >88 cm en mujeres.^15^. En cie2 se utilizarán los valores de corte obtenidos empíricamente en adultos argentinos para IMC y cc: en varones IMC ≥ 28,6 kg/M^2^ más cc ≥ 94,5 cm; en mujeres IMC ≥ 27,8 kg/m^2^ más cc ≥ 93,5 cm 14. Para CIE se decidió utilizar cc en lugar de ICT, ya que es lo sugerido en Argentina 6,15.

### Aspectos éticos

Por utilizar datos ya disponibles y de dominio público y por no existir ninguna posibilidad de identificar a los individuos de quienes se han recogido los datos aquí trabajados, el presente trabajo no representa riesgo alguno y no requiere mecanismos de control 16.

### Tratamiento de los datos

Se analizarán las variables IMC, ICT y cc y se identificarán a aquellas personas con CAE según los criterios ya señalados. Se calcularán prevalencias y se realizará estadística descriptiva de todas las variables. Se compararán los resultados según el sexo autorreportado de acuerdo al tipo de variable estudiada; para las variables dicotómicas se utilizará Chi cuadrado, mientras que para las variables cuantitativas dependerá del tipo de distribución que presenten. La significancia estadística se estableció en 95%. Los estadísticos se calcularán con el software IBM-SPSS v.20.

## RESULTADOS

De las 16 577 personas que formaron parte del Paso 2 (mediciones físicas) se retiraron 338 sujetos por falta de alguno/s de los datos antropométricos (peso, talla o circunferencia de cintura), tres sujetos por presentar valores antropométricos inconsistentes, dos sujetos menores de 18 años y 3363 sujetos mayores de 65 años, quedando 12 871 participantes de ambos sexos y de 18 a 65 años de edad para analizar.

En la Tabla 1 se muestra la prevalencia de CAE para la muestra total y para cada sexo según los diferentes criterios analizados.

**Tabla 1:** Estadística descriptiva del componente antropométrico para el diagnóstico de la obesidad en adultos argentinos de ambos sexos

	Mujeres (n=5 825)	Varones (n=7 046)	Total (n=12 871)	Diferencia entre sexos*
IMC Me(RIC) kg/m2	26,8(23,3-31,2)	27,4(24,1-31,3)	27,1(23,7-31,2)	P=0,000
ICT Me(RIC)	0,54(0,47-0,61)	0,55(0,49-0,61)	0,54(0,48-0,61)	P=0,019
CC Me(RIC) cm	88,0(77-99)	92(82-102)	90(79-100)	P=0,000
Componente antropométrico alto % (IC 95%)				
Criterio tradicional	30,9 (29,7-32,0)	32,7 (31,6-33,8)	31,8 (31,0-32-6)	Chi=4,8 p=0,028
EASO1	56,3 (55,0-57-6)	61,8 (60,7-63,0)	59,3 (58,5-60,2)	Chi=40,6 p=0,000
EASO2	47,5 (46,2-48,8)	49,7 (48,5-50,9)	48,7 (47,8-49,6)	Chi=6,2 p=0,013
CIE1	48,0 (46,7-49,3)	31,6 (30,5-32,7)	39,0 (38,2-39,9)	Chi=361 p=0,000
CIE2	31,8 (30,6-33,0)	33,5 (32,4-34,6)	32,7 (31,9-33,5)	Chi=4,1 p=0,042

IMC: índice de masa corporal; ICT: índice cintura/talla; CC: circunferencia de cintura; Me: mediana; RIC: rango intercuartil; IC: intervalo de confianza; EASO: Asociación Europea para el Estudio de la Obesidad, por sus siglas en inglés; CIE: comité internacional de expertos. *Las variables IMC, ICT y CC no se comportaron de manera normal (Kolmogorov-Smirnov p=0,000) y se utilizó el estadísticos U de Mann-Whitney.

## DISCUSIÓN

Las prevalencias obtenidas con cada criterio del CAE han presentado mayores o menores diferencias entre ellas, yendo desde un 31,8% de la población adulta argentina de ambos sexos con el criterio tradicional (1MO30) a un 59,3% con el criterio easo1 propuesto para población de ascendencia europea. El criterio ciE2, que utilizó valores de corte argentinos, fue el que más se asemejó al criterio tradicional, tanto en varones como en mujeres.

IMC y cc presentaron diferencias significativas entre varones y mujeres, ICT también aunque esas diferencias fueron menores. Esto parece sugerir que los valores de corte que se propongan deben diferenciar el sexo, tal como se sugiere en la literatura 9,17. Las prevalencias presentaron diferencias significativas aunque menores entre varones y mujeres, con la excepción de cie1, en el cual las mujeres superaron sustancialmente a los varones. Esto puede explicarse dado que cie1 presenta diferencias muy superiores a los otros criterios en cuanto a los valores de corte de cc entre varones y mujeres (véase Métodos) y señala la necesidad de revisar su utilización en población argentina.

Si bien alcanzar el CAE no es sinónimo de obesidad según los nuevos criterios, sí resulta ser parte necesaria para su diagnóstico o para asumir un riesgo de salud superior 8,9. Por este motivo, las diferentes prevalencias encontradas con cada criterio señalan que los procedimientos empleados pueden afectar a los resultados. En este caso, los procedimientos incluyen el criterio antropométrico por utilizar (EASO, CIE, otros), las características de la muestra, la técnica de medición antropométrica, la forma de seleccionar el valor de corte y el indicador de salud utilizado para establecer esos valores, entre otros 9,14,17. En este sentido, el CIE ha destacado la necesidad de nuevas investigaciones para identificar valores de corte más precisos y fiables y aclararon que proporcionarán directrices específicas (9, apéndice 2, p. 11).

Se concluye que será necesario trabajar sobre la identificación de cuál de los criterios utilizados en esta publicación, o algún otro que deberá definirse, representa mejor el exceso de adiposidad para la identificación de adultos argentinos con obesidad ♣
